# Mutant p53 in cell-cell interactions

**DOI:** 10.1101/gad.347542.120

**Published:** 2021-04-01

**Authors:** Steven Pilley, Tristan A. Rodriguez, Karen H. Vousden

**Affiliations:** 1 The Francis Crick Institute, London NW1 1AT, United Kingdom;; 2 National Heart and Lung Institute, Imperial College, Hammersmith Hospital Campus, London W12 0NN, United Kingdom

**Keywords:** cancer, cell competition, cell-cell interactions, mutant p53

## Abstract

p53 is an important tumor suppressor, and the complexities of p53 function in regulating cancer cell behaviour are well established. Many cancers lose or express mutant forms of p53, with evidence that the type of alteration affecting p53 may differentially impact cancer development and progression. It is also clear that in addition to cell-autonomous functions, p53 status also affects the way cancer cells interact with each other. In this review, we briefly examine the impact of different p53 mutations and focus on how heterogeneity of p53 status can affect relationships between cells within a tumor.

## Abstract (precis)

In this review, Pilley et al. examine the impact of different p53 mutations and focus on how heterogeneity of p53 status can affect relationships between cells within a tumor.

Genome-wide analyses have shown that *TP53* is the most frequently compromised gene in human cancer (Kandoth et al. 2013). In the 40 yr since p53 was discovered, its function has been studied intensively, yet new activities are still being found. Foremost, p53 is a transcription factor with the ability to control the expression of a large number of coding and noncoding RNA (Beckerman and Prives 2010; Grossi et al. 2016; Sullivan et al. 2018). Additionally, p53 interacts with cytoplasmic and mitochondrial proteins to directly modulate their activity (Ho et al. 2020). p53 sits as a central node in the cell's stress detection pathways, integrating signals from numerous sources, including DNA damage, oncogenic stress, hypoxia, and metabolic stress, to determine cell fate according to the type, severity, and duration of the stress. The induction of a p53 response can help cells deal with stress by supporting adaptation and survival or induce a permanent withdrawal of proliferative capacity through the induction of differentiation, senescence, or cell death (Kruiswijk et al. 2015). In either case, the induction of a p53 response can retard the evolution of a fully malignant cell. However, it is clear that there is still much to be learned about the exact mechanisms through which p53 fulfills its role as our most important defense against cancer development.

## Mutant p53

While some cancer-associated somatic mutations in *TP53* result in the loss of protein expression, many cancers express missense mutations that lead to the expression of full-length p53 proteins carrying a single amino acid substitution. Li-Fraumeni syndrome (LFS) patients carry germline p53 mutations and are consequently highly likely to develop a variety of different cancers at a young age (Bougeard et al. 2015). Most (∼80%) somatic missense mutations occur in the central DNA binding domain (DBD) of p53 and are concentrated at certain “hot spot” residues, including: 175, 245, 248, 273, and 282 (Bouaoun et al. 2016). These DBD p53 mutations have been classified as either affecting the conformation or DNA contacting ability of the protein, but most mutants show a reduction in both structural stability and sequence-specific DNA binding (Bullock et al. 2000), and it is not clear whether these classifications are functionally relevant (Sabapathy and Lane 2018). Missense mutations in the N-terminal domain and C-terminal oligomerization domain (OD), which flank the DBD of p53, are also found in human cancers, although somatic alterations in this region are less frequent. Interestingly, the R337H OD alteration is found in a significant number of LFS families in southern Brazil (Pinto and Zambetti 2020).

**Figure 1.**
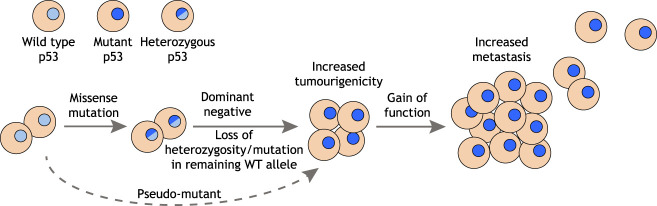
Acquisition and impact of mutant p53 status. Expression of a mutant p53 protein is associated with more aggressive behavior in many cancer types, and mutant p53 status may be acquired through several mechanisms. Following point mutation in *TP53*, cancer cells can become genetically homozygous or hemizygous through loss of the WT allele (loss of heterozygosity). However, in cells that retain WT p53, the dominant-negative activity of mutant p53 can inhibit the WT protein and render the cell functionally mutant for p53. Finally, the adoption of a pseudomutant p53 conformation by the WT protein can allow mutant p53 behavior in cells without a *TP53* mutation. Additional gain-of-function activities of mutant p53, beyond loss of WT activities, are associated with progression to metastasis in several cancer types.

Cancer-derived p53 missense mutants are impaired for most wild-type (WT) p53 functions. While this loss of function can clearly contribute to tumor development, the high incidence of missense mutation compared with nonsense mutation or gene deletion has raised the possibility that there is a selective advantage to tumors in maintaining the expression of mutant p53 proteins. Studies comparing the consequences of loss of p53 versus expression of mutant p53 have revealed a number of mechanisms that could explain the selective advantage of mutant p53 expression during cancer development. These can be broadly grouped into dominant-negative effects on coexpressed WT p53 or independent gain of oncogenic functions (Fig. 1).

Despite the vast literature identifying mutations in p53 as contributors to cancer development, a surprising study recently suggested that, in some contexts, mutant p53 could function as a tumor suppressor (Kadosh et al. 2020). Using a mouse model of intestinal cancer driven by the deletion of *CKIa* or mutation in *Apc*, mutant p53 was shown to have the expected oncogenic effect in the distal tissues of the intestine. However, in the proximal intestinal tissues of the same mice, the presence of mutant p53 was tumor-suppressive, a response that was shown to result from differences in the local microbiome. These results were quite unexpected, as such clear regional differences between mutant and loss of p53 have not been noted in other studies. The critical factor necessary to support mutant p53's oncogenic activity was found to be microbiota-derived gallic acid, leading to the possibility that changes in gallic acid levels between different mouse colonies could influence mutant p53-driven tumorigenesis. Nevertheless, while this tumor suppressor role for mutant p53 clearly warrants further investigation, almost all of our understanding of mutant p53 function to date addresses its protumorigenic activities.

### The dominant-negative effect

**Figure 2.**
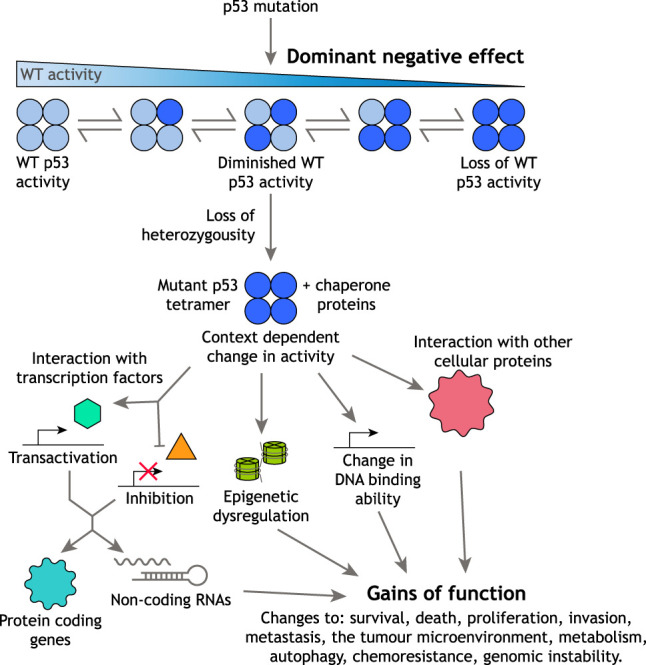
Mechanisms of mutant p53 action. The acquisition of p53 mutation can lead to the formation of heterotetramers made up of WT and mutant monomers. The presence of mutant subunits in a tetramer can reduce WT p53 activity and, through this dominant-negative activity, allow for tumor development. However, many tumors lose the remaining wild-type allele, leading to the expression of only mutant protein. Mutant p53 is frequently stabilized in cancer cells, in part as a consequence of complex with chaperone proteins, allowing it to mediate a variety of new activities (gains of function) via a number of different mechanisms.

p53 functions as a tetramer, assembled through the C-terminal OD. This region is retained by the common cancer-associated point mutants of p53, allowing the formation of functionally compromised heterotetramers containing both WT and mutant proteins. A dominant-negative (DN) ability of mutant p53 to restrain the activity of WT p53 could contribute to tumor development when both are expressed in the same cell (Fig. 2). In established tumors, estimates of the frequency of a loss of heterozygosity (LOH) in *TP53* (the deletion of the WT *TP53* allele after mutation in the other) vary between cancers. However, a recent analysis of >10,000 tumors reported that >90% of tumors with *TP53* mutations did not retain a WT allele (Donehower et al. 2019). The frequent loss of the WT allele may indicate that the DN activity of mutant p53 proteins is not sufficient to fully inhibit WT p53 activity and allow for the development of tumors (Alexandrova et al. 2017). Furthermore, an analysis of several tumor types failed to show any difference in the rate of LOH in tumors expressing mutant p53 with DN capacity compared with those with p53 mutant proteins unable to tetramerize and so lacking DN function, or in the survival of patients harboring these tumors (Shahbandi and Jackson 2019). On the other hand, analysis of cancers arising in LFS patients—with a germline mutation in one *TP53* allele—showed loss of the WT allele in almost all cases with a p53-null mutation but in only around a third of tumors in LFS patients carrying a germline p53 mutation in the DBD (Varley et al. 1997; Varley 2003; Malkin 2011). LFS patients carrying a potentially DN p53 allele also showed an earlier tumor onset than those with functionally null p53 mutations (Bougeard et al. 2015).

To seek clarity around the role of DN functions of mutant p53, several experimental models have been used. Although the DN activity of mutant p53 has been demonstrated repeatedly over the past 30 yr, many of the early studies used systems that overexpressed the mutant protein and may not reflect a physiologically relevant situation (Gencel-Augusto and Lozano 2020). Nevertheless, more recent analyses of cells or mice expressing endogenous mutant and WT p53 have demonstrated DN activity of the mutant protein under some, but not all, conditions (Gencel-Augusto and Lozano 2020). In mouse models, a substantial proportion of tumors arising in *Tp53*^+/−^ mice retain a functional WT allele (Venkatachalam et al. 1998). While it is possible that aging (Feng et al. 2007) and the acquisition of other alterations (such as defects in the pathways necessary to activate p53) weaken the p53 response in tumors that retain WT p53, it seems likely that complete loss of WT p53 function is not necessary for tumor development, so limiting a requirement for any DN effect. *Tp53*^*+/mutant*^ mice, in which the p53 mutant has potential DN activity, show extended survival compared with p53-null mice, again suggesting that the expression of mutant p53 cannot completely disable the tumor restraining functions of the WT protein (Lang et al. 2004). This concept is further supported by the analysis of the consequences of loss of the p53 regulator MDM2 in mice. Expression of MDM2 during embryogenesis is essential to restrain p53 activity, but the lethality of *Mdm2* loss can be rescued by concomitant deletion of both *Tp53* WT alleles. However, deletion of *Mdm2* in a *Tp53*^*+/mutant*^ mouse also led to embryonic lethality, indicating that WT p53 is still active (Lang et al. 2004).

While these mouse experiments show that the expression of mutant p53 cannot completely inactivate WT p53 function, several models show that some mutant p53s have a partial ability to restrain WT p53. Several studies have reported that the presence of a mutant p53 allele can result in a decrease in WT p53 functions, mostly in response to various forms of DNA damage (Lang et al. 2004; Wijnhoven et al. 2007; Lee and Sabapathy 2008). Importantly, p53 protein can only be detected in tumor samples and not in normal tissue of *Tp53*^*+/mutant*^ mice (Lang et al. 2004; Olive et al. 2004), suggesting that there are some tumor-specific changes required for mutant p53 to become stable and therefore able to exert a DN effect. Several mechanisms may underlie the stabilization of mutant p53 in cancer cells, including: the presence of tumor-specific stress signals that might normally function to stabilize WT p53, the inability of most mutant p53 proteins to induce the transcription of MDM2, and the ability of mutant p53 proteins to complex with members of the heat shock protein family, induced by the unfolded protein response often active in tumors, which stabilize mutant p53 proteins by preventing interaction with MDM2 (Wawrzynow et al. 2018; Mantovani et al. 2019). Furthermore, by disrupting the autophagic machinery in the cell, mutant p53 protects itself from degradation, adding to its stability (D'Orazi et al. 2020). Taken together, it appears that, in most cases, mutant p53 has only a partial ability to limit WT p53 function and that this may only become relevant in certain tissues or in response to stress such as DNA damage (Gencel-Augusto and Lozano 2020). A more in-depth analysis of studies that have or have not found evidence for the DN effect can be found in this recent review (Gencel-Augusto and Lozano 2020).

### Oncogenic gains of function

In addition to inhibiting the function of WT p53, mutant p53 proteins can also acquire novel gains of function (GOFs) that contribute to tumor development independently of WT p53. The most compelling evidence for mutant p53 GOFs is seen by comparing mice expressing mutant p53 or no p53. While p53-null mice develop mostly lymphomas and soft tissue sarcomas (Donehower et al. 1992), mice expressing mutant p53 show a different tumor spectrum; developing more epithelial and endothelial tumors (Olive et al. 2004) and showing increased metastasis (Lang et al. 2004). Similarly, mice expressing a humanized version of mutant p53 show worse survival than mice without p53 (Hanel et al. 2013). A positive role for mutant p53 in maintaining malignancies has also been shown in cell culture systems in vitro (Bossi et al. 2006; Hui et al. 2006) and in vivo, where inactivation of mutant p53 caused tumor shrinkage and improved survival in lymphomas (Alexandrova et al. 2015) and tumor shrinkage in dextran sulphate sodium (DSS)-induced colorectal cancer (Schulz-Heddergott et al. 2018).

Further experimental studies have revealed a variety of cell-autonomous functions of mutant p53s, including cell survival, chemoresistance, metabolic rewiring, inhibition of autophagy and cell death, resistance to proteotoxic stress, increased rates of proliferation, genomic instability, and enhanced migratory and invasive capacity. These activities are discussed in detail in several recent reviews (Freed-Pastor and Prives 2012; Muller and Vousden 2014; Kim and Lozano 2018; Sabapathy and Lane 2018; Stein et al. 2019b; Zhang et al. 2020a; Zhu et al. 2020). In general, these functions reflect the acquisition or alteration of DNA and protein binding abilities of mutant p53s. While tumor-associated p53 mutant proteins lose most, but not necessarily all, of their ability to bind to the consensus DNA binding sequences recognized by WT p53 (Ludwig et al. 1996; Kato et al. 2003), they can acquire the ability to bind different promoters and so directly regulate new transcriptional programs (Dell'Orso et al. 2011). However, most of the GOF activities displayed by different mutant p53 proteins are thought to manifest through their interactions with other proteins. Mutant p53 proteins have been found to interact with a number of different transcription factors—such as NF-Y, SREBPs, ETS1/2, and NF-κB—and either potentiate or inhibit their activity (Kim and Lozano 2018). In particular, mutant p53s bind to and suppress the activities of the family member transcription factors p63 and p73 (Strano 2016). These mutant p53/transcription factor interactions lead to changes in expression of both protein coding genes and a range of different noncoding RNAs, including miRNAs and lncRNAs (Zhang et al. 2020a), all of which can contribute to the GOF phenotype. The activity of mutant p53s is further expanded by the ability to bind and modulate the activity of nontranscription factor proteins, resulting in the impairment of the DNA damage response, increased rates of glycolysis and lipid production (Zhu et al. 2020), and increased histone methylation (Chen et al. 2019). More generally, mutant p53s can bind to a variety of proteins controlling the epigenome, including those that regulate chromatin structure, histone modifications, and splicing, resulting in genome-wide dysregulation (Escobar-Hoyos et al. 2020; Zhang et al. 2020a). It should be noted, however, that the majority of the outcomes of mutant p53 expression are highly mutant- and cell type-specific, suggesting that the particular external stress and the composition of the tumor microenvironment (TME) may influence mutant p53 GOFs (Fig. 2; Amelio and Melino 2020).

### Resolving the roles of mp53 DN activities and GOFs

There is considerable debate about the importance of DN and GOF activities of mutant p53 (Stein et al. 2020). An analysis of genome-wide RNAi and CRISPR-Cas9 survival screens of cancer cell lines did not reveal the dependence of cancer cells on mutant p53 expression, although this study did show that many p53 point mutants acquire a DN ability to overcome WT p53 activation and allow proliferation and survival of cells in culture (Giacomelli et al. 2018). Similarly, engineering of AML cell lines to express various combinations of WT and mutant p53 provided evidence for DN but not GOF activities. However, in vivo hematopoietic stem and progenitor cells (HSPCs) null for p53 outcompeted HSPCs expressing one mutant p53 and one WT allele in response to sublethal radiation, indicating the limitations of the DN effect (Boettcher et al. 2019). In a system lacking WT p53, an analysis of >10,000 p53 mutants found evidence for GOFs, with cells expressing mutant p53s gaining an in vivo advantage over cells without p53 (Kotler et al. 2018). Furthermore, expression of mutant p53 enhances the reprogramming and transformation of somatic cells (Sarig et al. 2010) and drives abnormal self-renewal in acute myeloid leukemia beyond that seen following p53 loss, again supporting a role for mutant p53 beyond loss of WT function (Loizou et al. 2019).

Collectively, these studies leave a confusing picture. There is good evidence for both GOFs and DN activities, but neither are consistently observed in all models, and it is clear that the manifestation of these phenotypes will be context- or tumor type-specific. It is worth considering that the temporal dynamics of LOH in *TP53* in cancers are not well established, meaning DN activity may be important during tumor initiation but not in the later stages of tumor progression, at least in some cancers. While it may be possible for GOFs to occur in the presence of the WT protein, in certain contexts, GOFs cannot be identified unless LOH has occurred (Nakayama et al. 2020). On the other hand, GOFs may become more important in driving the metastatic progression of established malignancies.

Adding to the complexity are intriguing studies showing that mutant p53s can form prion-like aggregates that also capture p53 family members and may underlie both DN and GOF activities. When internalized by otherwise healthy cells, mutant p53 aggregates coaggregate with and consequently inhibit WT p53 protein activity, suggesting that mutant p53 status can spread between cells (de Oliveira et al. 2020; Navalkar et al. 2020).

### WT p53 in tumorigenesis—the importance of structure

Whereas mutations in p53 are a common occurrence in cancers, it is clear that tumors can develop while retaining WT p53. The identification of mechanisms through which p53 promotes cell survival in the face of mild stresses, such as nonlethal DNA damage or transient nutrient starvation, provides for the possibility that WT p53 functions may help to support some stages of tumor development. Such activities explain an early paradoxical observation that WT p53 activity could support the development of carcinogen-induced skin papillomas in mice (Kemp et al. 1993) and several subsequent studies showing that retention of WT p53 can promote resistance to therapy (Ablain et al. 2016; Webster et al. 2020). Furthermore, WT p53 in tumors has been shown to maintain cell survival in response to the depletion of nutrients such as glucose (Jones et al. 2005), serine (Maddocks et al. 2016), or glutamine (Tajan et al. 2018) by promoting the metabolic plasticity required to deal with the stress. Some tumor-derived p53 mutants retain the ability to deal with these stresses, suggesting that a retention of some WT p53 activities by mutant p53 can benefit tumors (Tran et al. 2017; Humpton et al. 2018).

Interestingly, expression of a p53 protein with WT sequence does not always ensure the retention of WT p53 structure or function. WT p53 can adopt a “mutant” conformation under some conditions, such as defects in chaperones, leading WT p53-expressing cells to display mutant p53-like functions such as increased invasion (Trinidad et al. 2013) and gene expression patterns (Benor et al. 2020). The expression of this “pseudomutant” phenotype may explain how some cancers develop despite the retention of WT p53 and suggest that some plasticity with respect to WT and mutant p53 activity may be highly beneficial during tumorigenesis.

Notwithstanding the potential for some WT p53 functions to support cancer development, there is abundant evidence to support the therapeutic benefit of WT p53 activation in many cancers (Lozano 2019). Consequently, an approach that allows for the reactivation of WT p53 function in tumors expressing mutant p53 proteins is an attractive therapeutic strategy. A variety of different compounds have been tested for their ability to restore the WT conformation and consequently the function of mutant p53 proteins (Muller and Vousden 2014). These include APR-246, a prodrug reported to restore WT conformation by binding specific cysteine residues, which is currently in clinical trials. However, APR-246 and its related compounds also show a variety of p53-independent activities that could underlie therapeutic efficacy (Bykov et al. 2018; Eriksson et al. 2019).

## Cell-nonautonomous roles of p53

It has become clear that, to fully understand cancer, the tumor cells need to be considered in the wider context of the TME. Cancer cells (of which there may be various subclones) make up only a fraction of a solid tumor. The rest of the tumor mass comprises the stroma, consisting of a variable proportion of different immune cells, fibroblasts, adipocytes, blood vessels, and nerves. This composition has led to the description of tumors as “pseudoorgans” (Lyssiotis and Kimmelman 2017). While competition between these cells for space and resources imposes challenges on tumor cells, surrounding stromal cells can also be reprogrammed by cancers to provide support and enhance malignant development. Most previous studies on p53 have focused on cell-autonomous functions of WT and mutant p53, but it is becoming clear that the complex interaction between cancer cells and their surroundings is also strongly influenced by the p53 status of each compartment.

### WT p53 in cell-nonautonomous tumor suppression

Several studies have shown that loss of WT p53 in normal stromal cells enhances tumorigenesis (Kiaris et al. 2005; Guo et al. 2013, 2017), demonstrating a tumor-suppressive role for p53 beyond the cancer cells themselves. This activity is linked to the ability of WT p53 to activate expression of various secreted factors that can limit oncogenic progression (Fischer 2017). Examples include an ability of p53 in fibroblasts to exert a tumor-suppressive effect on the surrounding tissue by suppressing the expression of SDF-1/CXCL12, a chemokine that increases cell invasiveness (Moskovits et al. 2006) and the release of factors from p53-expressing hepatic stellate cells that promote macrophage polarization toward the tumor-suppressive M1 type (Lujambio et al. 2013). The induction of senescence in stromal cells following activation of p53 is associated with the release of cytokines and other factors related to the “senescence-activated secretory phenotype” (SASP). The consequences of SASP induction can be both tumor-promoting and tumor-limiting (Coppé et al. 2010), and the promalignant activities of SASP have been shown to be reduced by p53 (Coppé et al. 2008). Further evidence is now developing to show a function of WT p53 in other types of stromal cells, such as immune cells (Blagih et al. 2020a), with variable effects on tumorigenesis.

Although stromal cells can provide tumor-limiting functions, it is well established that cancer-associated fibroblasts (CAFs) exhibit activities that promote tumor growth and progression (Kalluri and Zeisberg 2006). Interestingly, cancer cells can dampen the tumor-limiting responses driven by p53 in surrounding fibroblasts by inhibiting their ability to activate p53 (Bar et al. 2009). In a similar model, p53 suppression or mutation in tumor cells was also seen to reduce p53 activity in cocultured fibroblasts and also stimulated fibroblast proliferation and their tumor-supporting functions via miRNAs found in exosomes released by the tumor cells (Yoshii et al. 2019). Loss of p53 in fibroblasts has previously been associated with their transformation into tumor-promoting CAFs (Addadi et al. 2010). Furthermore, p53 was found to be critical in supporting the expression of genes that distinguish CAFs from normal fibroblasts, including those of secreted proteins that increase cancer cell migration and invasion in vitro and growth in vivo (Arandkar et al. 2018). The investigators explain that tumor cells acquire the ability to inhibit the canonical tumor-suppressive functions of p53 in normal fibroblasts, and in CAFs, the p53 activity becomes “rewired” to support their tumor-promoting functions (Arandkar et al. 2018). Further investigations into how and why the functions of WT p53 switch from tumor-suppressive to tumor-promoting are required, although it is possible that the “pseudomutant” phenomenon may be responsible. WT p53 has also been shown to promote chemoresistance through a cell-nonautonomous manner by enabling cells to enter into senescence. While mutant p53 cells die due to their inability to arrest proliferation, WT p53 senescent cells secrete growth-stimulating cytokines in an autocrine and paracrine manner, allowing these tumors to relapse faster after the withdrawal of chemotherapy (Jackson et al. 2012).

The retention of WT p53 in cancer cells can also affect the tumor-stroma interaction, most clearly by supporting the activation of an anti-tumor immune response (Bezzi et al. 2018; Wellenstein et al. 2019; Blagih et al. 2020b). In these studies, loss of p53 in cancer cells was shown to increase expression of cytokines and factors that blunt various arms of the immune response to the tumor and enhance metastasis. Finally, p53 has also been shown to restrain the adrenergic transdifferentiation of neurons in murine oral tumors by promoting the release of vesicles containing the microRNA miR-34a. These changes in innervation resulting from p53 loss contributed to cancer progression in mice and were correlated with a significant worse prognosis in human cancers (Amit et al. 2020).

### Cell-nonautonomous functions of mutant p53

As with WT p53, the presence of mutant p53 in a cell changes the expression of a variety of secreted proteins in a context- and mutation-dependent manner. The profile of molecules secreted by a tumor cell depends on the tumor genetics (Paltridge et al. 2013) but also varies widely based on the cancer type (Robinson et al. 2019). The impact of mutant p53 on the cancer cells secretome and consequent interactions between the tumor and stroma have been discussed in detail in several previous reviews (Cordani et al. 2016; Stein et al. 2019a; Blagih et al. 2020a; Pavlakis and Stiewe 2020; Pavlakis et al. 2020) and is briefly reviewed here.

Changes in the proteins that are secreted by cancer cells can have a direct effect on their behaviors through autocrine or paracrine mechanisms, and the induced expression of a variety of p53 mutants including R175H, R248Q, R248W, R249S, R273H, and R282W in H1299 cells led to the expression of a proinvasive secretome that increased the invasiveness of cells with no p53 expression (Neilsen et al. 2011). Mutant p53 was also shown to enhance exosome-mediated Hsp90a secretion (Zhang et al. 2020b), a protein that promoted invasion and migration of cancer cells and was previously shown to increase the efficiency of metastasis in mice (Wang et al. 2009). In addition to the up-regulation of specific secreted factors, mutant p53 has also been found to amplify total cell secretion by inducing the transcription of *miR-30d*, which is able to influence the structure of the Golgi apparatus. The mutant p53-dependent increase in secretion enhanced tumorigenesis and metastasis in vivo (Capaci et al. 2020).

A number of studies have identified how mutant p53 modulates the response of the NF-κB pathway to inflammatory signals, affecting the cellular secretions and consequently tumor cell motility and survival. For example, expression of various p53 mutants (including R175H, M237I, R273H, and R280K) supported invasive behavior and cancer cell survival in response to the inflammatory cytokine TNFα by promoting the secretion of proinvasive molecules such as MMP9 and CXCL10. Interestingly, these cells also showed mutant p53-dependent secretion of the lymphocyte-attracting chemokines CX3CL1 and LTB, suggesting that the increased motility was accompanied by an induction of a potentially tumor-limiting immune response (Di Minin et al. 2014). Similarly, several different mutant p53 proteins (D281G, H179L, R175H, and R273H) were shown to cooperate with the NF-κB pathway to promote CXC chemokine expression to increase cell motility over p53-null cells (Yeudall et al. 2012). The mutant p53-induced production of cytokines has also been shown in several studies to prevent the anti-tumor immune response and drive a protumoringenic inflammatory environment (Agupitan et al. 2020).

Another area of growing interest is how mutant p53 affects the production of exosomes by cancer cells can impact tumor progression. Exosomes are small extracellular vesicles that can contain nucleic acids, proteins, lipids, and metabolites, allowing communication between cells and tissues (Kalluri 2016). Exosomes secreted by mutant p53-expressing colon cancer cells can reprogram adjacent macrophages into a “cancer-promoting” state, making them more able to support tumor growth and metastasis than macrophages exposed to WT p53 cancer cells.(Cooks et al. 2018). Enhanced production of exosomes by mutant p53-expressing cancer cells also leads to an increase in fibroblast mobility and educates fibroblasts to deposit and remodel the extracellular matrix (ECM) into a state more conducive to cancer cell invasion (Novo et al. 2018).

Mutant p53 had previously been shown to play a role in modifying the structure of the ECM through the retention of the WT ability to repress the expression of TIMP3, an inhibitor of the matrix metalloproteinases (Loging and Reisman 1999), the activation of which results in the degradation of the surrounding ECM, consequently enhancing metastasis and invasion (Kessenbrock et al. 2010). Furthermore, MMP9 is expressed at much higher levels in colorectal cancers with p53 mutations than in those without (Rahnamoun et al. 2017). In nonsmall cell lung cancer, interactions between mutant p53 R273H HIF1α result in the up-regulation of ECM proteins VIIa1 collagen and laminin-γ2, the expression of which is associated with worse prognosis (Amelio et al. 2018). Another investigation found mutant p53 in tumor cells can have an indirect effect on the ECM by affecting fibroblast secretions. Mutant p53-expressing pancreatic cancer cells induced the secretion of perlecan in adjacent fibroblasts, an activity that was required for invasion of both mp53- and p53-null cancer cells into matrices (Vennin et al. 2019). The ability of mutant p53 to affect the structure of the ECM may have a feedback effect on the tumor cells, since mutant p53 is known to affect the trafficking of receptors that interact with the ECM, such as integrins (Muller et al. 2009), and become stabilized in response to changes in matrix stiffness (Ingallina et al. 2018).

Tumor-stromal interactions involve cross-talk between multiple different cell types in the TME, where an effect on one stromal compartment can have a subsequent impact on another. Not surprisingly, the p53 status of the tumor can influence these complex interaction networks. For example, tumor cells can induce fibroblasts to secrete IFNβ, which represses the invasive activity of the cancer cells and has potential to modulate the immune response to the tumor. The response to CAF-derived IFNβ is modulated by mutant p53 expression in the cancer cells, leading to increased invasive capacity (Madar et al. 2013).

#### Heterogeneity in the impact of mutant p53 on the stroma

There is a growing appreciation that different p53 mutants may have different activities that support and promote cancer development (Hanel et al. 2013; Giacomelli et al. 2018; Sabapathy and Lane 2018), an observation that also extends to the functions of p53 in controlling the stromal interaction. The ability to drive expression of secreted proteins that enhance malignancy, such as CXC chemokines, proinflammatory cytokines, and extracellular matrix-related proteins, is stronger in the “DNA contact” mutants R248Q and R273H than “conformational” mutants R175H and H179R, with some evidence that these two groups of p53 mutants function through different pathways (Buganim et al. 2010). In contrast, expression of the G245S mutant did not alter the expression of protumorigenic factors (Solomon et al. 2012). Similarly, the ability of mutant p53 to traffic Hsp90a into exosomes was more robust for the DNA contact mutants (R248W, R273H, and R280K) than conformational mutants (R175H and Y220C) (Zhang et al. 2020b). This function of mutant p53 contributes to enhanced invasion, although, interestingly, the conformational mutants such as R175H are as effective in driving increased invasive capacity as the DNA-contacting mutant R273H (Zhang et al. 2020b). Similarly, the ability of cancer cells to induce changes in macrophages was also shown to be dependent on the type of p53 mutation in the tumor cells, with R273H and R249S, but not p53 V157F and R175H, able to induce a tumor-promoting shift in the profile of M2 macrophages (Cooks et al. 2018).

### Cell competition

The accumulation of postzygotic changes in DNA ensures that no two cells in an individual are likely to be genetically identical (Forsberg et al. 2017). In tumors, this genetic heterogeneity is further amplified by increased levels of DNA damage and enhanced still further by factors such as epigenetic, metabolic, and positional diversity (Martincorena and Campbell 2015). Evolutionary pressures driven by tumor progression and therapeutic intervention ensure that the complexity within the cancer cell population also changes over time (Caswell and Swanton 2017). Consequently, tumors are composed of ever-changing subpopulations of cells that differ from each other, establishing a situation where cells can compete with each other for survival and space.

First identified in *Drosophila*, cell competition describes the ability of cells to compare their fitness levels with that of their neighbours. During cell competition, cells carrying mutations that do not directly affect their viability in a tissue or tumor become disadvantaged when surrounded by fitter “winner” cells and are actively eliminated (Bowling et al. 2019). Various mechanisms can determine how differences in fitness levels between cells are sensed, and the elimination of the less fit “loser” cells can occur through a variety of different processes, including senescence, apoptosis, extrusion from an epithelial layer, or entosis. Finally, winner cells can compensate for the elimination of the loser cells by increasing proliferation or cell volume (Bowling et al. 2019; Baker 2020).

While much cell competition research has been conducted in nontumor developmental scenarios, there is clear potential for a tumor-suppressive role for cell competition in maintaining tissue homeostasis, ensuring that normal patterns of growth and development are not overrun by damaged or malignant cells. Several examples have been identified in flies and mammals, including “epithelial defense against cancer,” the process in which transformed cells are identified and eliminated in epithelia cell layers (Tanimura and Fujita 2020). However, cell competition can also be hijacked by tumor cells to allow them to outcompete normal cells in a process known as “supercompetition.” The complexity of competition between cells expands as tumors evolve, with likely ongoing competition between less and more aggressive clones. Emerging evidence also suggests the potential for cancer cells to compete with surrounding stromal cells. Given the clear potential for cell competition in modulating tumor development, it is not surprising that there is now a growing body of research identifying a role for p53 in this process.

#### A role for p53 in the losers during development and in normal tissue

**Figure 3.**
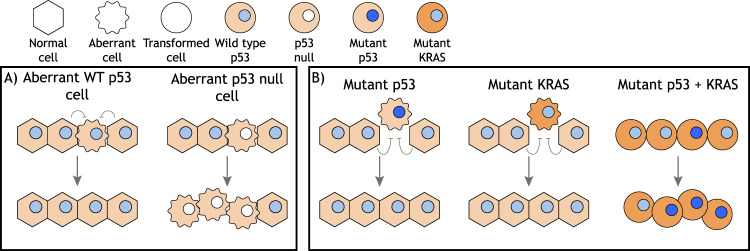
A role for p53 in cell competition. (*A*) Several studies have shown that WT p53 is required in aberrant cells that are outcompeted by surrounding cells containing WT p53. Loss of p53 allows the expansion of the aberrant clone. (*B*) In other contexts, cells expressing mutant p53 protein are outcompeted by WT p53-expressing cells and eliminated from epithelial layers. A similar loser phenotype is displayed by mutant KRAS-expressing cells. However, in the context of a mutant KRAS-expressing epithelial layer, cells with a concurrent mutation in *TP53* are not outcompeted.

During embryogenesis, the high mutation rate results in an increased chance that faulty cells may arise (Ju et al. 2017). Cells carrying defects such as abnormal ploidy or loss of genes crucial to patterning can persist if all the cells in the embryo are subject to the same defect. However, if the defective cells are surrounded by WT cells, then they will be eliminated by cell competition (Sancho et al. 2013), preventing them from propagating and forming part of the germline. Several studies have shown that p53 activity can track with the elimination of loser cells during development (Fig. 3A). An unbiased search for genes that controlled cell competition in mice showed that cells lacking p53 were able to outcompete cells retaining WT p53 in the embryo (Dejosez et al. 2013). Similarly, elimination of embryonic stem cells mutant for the BMP receptor *BMPR1A* depends on p53 in the losing cells. In this case, p53 was shown to signal a reduction in mTOR activity, leading to reduced fitness (Bowling et al. 2018). Consistently, cells with mildly increased p53 levels driven by reduced activity of MDM2 and MDM4, the key regulators of p53 stability and activity, were outcompeted by WT cells in mosaic embryos (Zhang et al. 2017). Interestingly, the fate of the p53-driven loser cells could be apoptosis (Bowling et al. 2018) or reduced proliferation (Zhang et al. 2017), suggesting that the response to p53 may differ depending on context.

A role for p53 as a mediator of reduced fitness has also been seen in damaged or mutant cells, where limited activation of p53 (insufficient to promote cell-autonomous cell death) plays an important role in marking cells as losers (Fig. 3A). For example, in the mouse hematopoietic stem cell niche, mildly damaged cells are outcompeted by healthy cells and enter senescence, a response that is dependent on p53 in the damaged, loser cells (Bondar and Medzhitov 2010; Marusyk et al. 2010). The elimination of tetraploid cells by diploid cells during embryogenesis is also dependent on p53 (Horii et al. 2015). Similarly, epithelial cells from normal canine kidney (MDCK cells) depleted of the tumor suppressor gene *SCRIB* (orthologous to the *Drosophila* gene Scribble) are outcompeted by WT cells as a consequence of direct, mechanically induced competition. Again, elevated levels of p53 were found to be inducing elimination of the loser cells, in this instance via a ROCK-p38 mechanical stress sensing pathway (Wagstaff et al. 2016).

#### Cell competition in suppressing and enhancing tumorigenesis

Mutations in oncogenes and tumor suppressors can have varied effects on cell fitness, with some oncogenic changes increasing competitive advantage while others lower fitness. In several cases, loss of tumor suppressors or gain of activated oncogenes can promote elimination of cells by competition. A number of genes identified in *Drosophila* to be critical for maintaining competitive cell fitness, such as *scrib* and *lgl*, are orthologs of human tumor suppressor genes (Menéndez et al. 2010), and in *Drosophila*, as in humans, mutations in these genes can promote the development of neoplasia. The loss of fitness associated with these mutations and the elimination of cells carrying these defects by surrounding normal cells points to an effective additional tumor suppressor function of cell competition. Similarly, in mammalian systems, cells acquiring oncogenic alterations such as KRAS, SRC, or YES activation can be outcompeted by WT cells, while loss of tumor suppressor proteins SCRIBBLE or VPRBP confers loser status to cells when in competition with WT neighbors (Tanimura and Fujita 2020), consistent with a tumor-suppressive role of competition. In vivo, the removal of oncogenic KRAS-expressing cells from WT tissue has also been shown in mouse skin and intestinal epithelium (Brown et al. 2017; Kon et al. 2017).

However, not all oncogenic alterations result in competitive disadvantage. One of the most studied regulators of competition is MYC, a family of proteins that is misregulated and contributes to the development of many cancers. In both flies and humans, overexpression of MYC results in the acquisition of a “superfit” phenotype, enabling these cells to outcompete surrounding normal cells by inducing their elimination (Paglia et al. 2020). Subsequent studies showed that loss of the tumor suppressors APC and HIPPO can also induce this “superfit” status (Vermeulen et al. 2013; Mamada et al. 2015), although these effects may reflect the ability of these alterations to drive the downstream activation of MYC. Interestingly, in humans, high MYC tumor cells also correlate with the induction of cell death in surrounding stromal cells (Di Giacomo et al. 2017).

Interestingly, the tumor-suppressive cell competition response can be overridden by the simultaneous acquisition of oncogenic alterations, as described below. This feature is reminiscent of the oncogene cooperation in the transformation of primary cells, described some 40 yr ago (Land et al. 1983). Taken together, it seems likely that cells of increasing aggressiveness will be selected, in part, for their ability to outcompete normal or less aggressive neighbors within the shifting heterogeneous tumor mass.

#### A role for p53 in tumor cell competition

Selection for mutations in p53 can be seen to occur at different stages of tumor progression in different cancers (Rivlin et al. 2011), indicating that their effect on cell competition could be varied and context-dependent. Furthermore, the details of the consequences of loss of WT p53 activity or acquisition of the expression of a mutant p53 protein on cell competition have not been clarified. Such considerations are complicated by the observation that in *Drosophila*, WT p53 is required for Myc-overexpressing cells to become supercompetitors (De La Cova et al. 2014), an activity that might predict loss of p53 to be tumor-suppressive. However, a similar requirement for p53 to support the supercompetitor phenotype in mammalian cells has not been described.

More easily understood is a role for cancer-associated mutations or loss of p53 to lead to a failure to eliminate loser cells, as seen during development and in normal tissue. Indeed, the role of p53 in allowing elimination of defective cells during development has prompted the suggestion that the persistence of these defective cells in p53-null mice, where competitive elimination of these cells by WT cells cannot occur, explains the development of lymphomas and soft tissue sarcomas so early on in their lives (Bowling et al. 2019). However, in cancers, most p53 mutations occur within the context of a WT individual, conditions in which cell competition will play a role in regulating tumor progression. Recent large scale sequencing studies have shown that apparently normal tissue harbors large numbers of cells carrying known oncogenic alterations, the frequency of which increases with age (Martincorena et al. 2015, 2018). How these potentially malignant cells are held in check is unknown, but expansion of these mutant clones in older individuals suggests that these cells may have a competitive advantage over surrounding WT cells. Intriguingly, the second most common alteration seen in these quasi-normal cells in the esophagus was mutation in p53 (Martincorena et al. 2018). In contrast, in some tissues, mutant p53 clones appear to accumulate only later in cancer development. Overall, it would seem that conditions for competition between normal and tumor cells of different p53 status may occur at any point throughout the course of tumor progression, although it is likely that the magnitude and mechanisms of these effects will depend on the cell type and tissue context.

The mouse epidermis has proven to be a useful model in which to study the role of p53 in cell competition. Upon division, daughter keratinocytes in the basal layer of the epithelial membranes can either retain their ability to proliferate or become terminally differentiated and migrate to the subrabasal layers. The mosaic introduction of mutant p53-R245W (equivalent to R248W in humans) into basal epithelial cells in mouse skin reduced the generation of differentiated daughter cells following keratinocyte division, maintaining a pool of proliferating cells that continued to expand at the expense of their WT neighbors. Interestingly, the resultant increased epidermal thickness and higher cell density in the basal layer of the epidermis was slowly restored to normal once the entire basal layer had been populated with mutant cells, suggesting the imbalance in cell fate had returned to normal (Murai et al. 2018). In response to UV irradiation, mutant p53 clones initially showed a more rapid expansion than WT clones, although continued UV exposure caused the mutant p53 population to regress, possibly because the acquisition of further mutations by WT cells allowed them to outcompete the mutant p53-expressing cells (Murai et al. 2018). Interestingly, other studies have suggested that the expansion of mutant p53-expressing clones of keratinocytes is restrained in normal epithelium but released in response to UV exposure, which allows the mutant p53-expressing clones to expand (Brash et al. 2005).

In the mouse intestine, KRAS activation or APC loss, introduced via low level recombination into Lgr5+ stem cells in the base of crypts, gives cells a competitive advantage over WT progenitor cells; in this case, the advantage of KRAS-expressing cells is seemingly in contrast to the loser phenotype seen following KRAS activation in other contexts as described above. However, acquisition of the p53 mutation R172H (equivalent to R175H in humans) was only seen to confer an advantage to cells after the induction of colitis by the administration of DSS, potentially reflecting the ability of p53 mutant cells to deal with colitis-associated reactive oxygen species (ROS) (Vermeulen et al. 2013).

A similar role of ROS has been seen in murine esophageal epithelium, where exposure to low levels of ionizing radiation (IR) drives the differentiation of cells due to an increase in oxidative stress. In this system, expression of mutant p53 made cells more resistant to ROS, thus protecting them against irradiation-induced differentiation and allowing for the expansion of the mutant, and potentially tumorigenic, clones (Fernandez-Antoran et al. 2019). The loser phenotype of normal cells in this context was rescued with antioxidant treatment, supporting the previous suggestion that mutant p53 increases cell fitness by helping cells to cope with increased ROS (Vermeulen et al. 2013).

Despite the broadly consistent ability of mutation in p53 to promote advantage in cell competition, studies on cultured cells have revealed that this can be context-dependent. p53-R273H-expressing MDCK cells were extruded from a monolayer by surrounding cells that were either WT or null for p53. Similar results were obtained from experiments performed with mouse intestinal organoids. Interestingly, however, if p53-R273H was sporadically induced within a monolayer of oncogenic RAS-expressing MDCK cells, there was no evidence of cell death or extrusion, suggesting that oncogenic RAS allowed the p53-R273H-expressing cells to escape the effects of the competition (Watanabe et al. 2018). Previously, the same lab reported that oncogenic RAS-expressing cells were extruded from WT epithelia (Hogan et al. 2009), indicating that epithelia possess a strong intrinsic tumor-suppressive mechanism protecting them from acquiring individual oncogenic events that can be overridden by certain combinations of alterations, such as KRAS activation and p53 mutation. Such considerations are likely to underlie the observation that activation of KRAS can promote loser or winner behavior in different experimental systems (Fig. 3B).

To date, analysis of the effect of mutation in p53 on interactions with surrounding WT cells has focused on p53 point mutations that give rise to mutant protein expression. As described earlier, these mutants generally lose WT p53 function but also have a potential to acquire dominant-negative or gain-of-function activities. Whether any p53 mutants show GOFs that support a “superfit” cell phenotype and how this might be modulated by the transformed state of tumor cells remains to be identified. The functions of WT or mutant p53 required to control cell competition are also not yet established, although the ability of various forms of p53 to control metabolite production, cytokine secretion, and the mechanical properties of the cell may all contribute to this activity.

#### Does mutant p53 affect competitiveness between tumor cells?

While interactions between WT and mutant p53-expressing cells are the most likely to occur during tumor development, it is also possible that tumors contain a mixture of cells that harbor different p53 mutations or with different levels of p53 expression. Immunohistological evidence shows heterogeneous levels of mutant p53 within tumors; for example, the Human Protein Atlas contains multiple images of tumor sections stained for p53, with clear evidence for profoundly different levels of p53 in individual cells in the tumor (https://www.proteinatlas.org/ENSG00000141510-TP53/pathology). The reasons for this heterogeneity are unclear, but there is evidence to suggest that mutant p53 expression patterns can be affected at both the gene and protein level.

p53 mutations acquired during tumorigenesis could lead to the formation of subclones with different p53 statuses within a tumor, resulting in areas with WT p53 and areas with no or mutant p53. Subclonal p53 mutations have been observed at varying frequencies in different cancer types, including lung (Jamal-Hanjani et al. 2017), skin (Albibas et al. 2018), and chronic lymphocytic leukemia (Nadeu et al. 2016), although a study of nine major cancer types showed that ∼95% of tumors are clonal for p53 mutations (McGranahan et al. 2015). These studies suggest that subclonality has a limited role as a driver of mutant p53 heterogeneity in tumors, although they may underestimate heterogeneity as a consequence of limited size of the tumor sample and sequencing depth (McGranahan et al. 2015). Furthermore, some tumors arise with more than one p53 mutation, potentially leading to a mixture of cells expressing different p53 mutants (Donehower et al. 2019; Gorelick et al. 2020). However, heterogeneity of mutant p53 expression in tumors is not necessarily a reflection of genetic differences in *TP53* in different subclones of cells. A study of human breast cancers showed that heterogeneity in mutant p53 protein levels was found in the majority of tumors (Bouchalova et al. 2014). Interestingly, variation in the mutant p53 levels was found to be dispersed randomly throughout the tumors and not localized to distinct areas, suggesting subclonal expansion to be an unlikely explanation.

**Figure 4.**
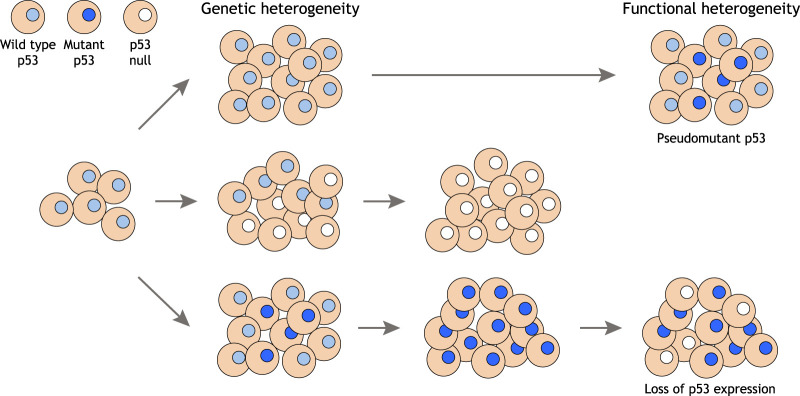
Heterogeneous p53 status in tumors. While some cancers retain a WT *TP53* gene, mutations in *TP53* occur during the development of many cancers, leading to expansion of clones of tumor cells that express no p53 or a mutant p53 protein among the WT p53-expressing cells. In some tumors, this heterogeneity of p53 expression persists, but most cancers tend to become clonal for cells carrying the *TP53* mutation, leading to genetic homozygosity with respect to *TP53.* However, signals that drive WT p53 into a pseudomutant conformation, or changes in adhesion characteristics within the tumor that result in loss of mutant p53 protein expression, can result in heterogeneity in p53 expression within a tumor that is clonal for the *TP53* gene.

Various explanations for this heterogeneous expression of mutant p53 have been put forward. Heterogeneity in the expression of genes that affect the proteasomal degradation of mutant p53, such as *MDM2* or *TRIM71*, may provide a genetic explanation for heterogenous mutant p53 expression (Koga et al. 2001), or changes in other systems that control the stability of mutant p53, such as association with chaperones or autophagy (as described above). Tumor cell differentiation has been proposed to lead to a drop in the transcription of *TP53*, causing a reduction in mp53 protein in some cells, and mp53 staining patterns may differ depending on the presence of the second WT p53 allele (Xue et al. 2019). IFNβ, which is expressed by stromal cells, can lead to the destabilization of mutant, but not wild-type, p53 mRNA, potentially leading to heterogeneity of mutant p53 expression (Madar et al. 2013). The stability of mutant p53 protein is also influenced by the stiffness of the surrounding extracellular matrix, through an ability of RHOA to prevent ubiquitination and degradation of mutant p53. Both genetic inhibition and a reduction extracellular matrix stiffness resulted in increased degradation of mutant p53 (Ingallina et al. 2018), leading to a situation where tumors carrying clonal p53 mutations can contain mixtures of cells that express high or low levels of mutant p53 protein, depending on local matrix availability. Similarly, tumors clonal for WT p53 may nevertheless contain mixtures of cells with WT and “pseudo”-mutant p53, where WT p53 adopts a mutant p53 conformation. An analysis of human breast tumors without *TP53* mutations found that some of them had transcriptional profiles that more closely resembled mutant p53 tumors than other WT p53 tumors. Furthermore, patients with pseudomutant p53 tumors show survival rates more similar to patients with mutant p53 tumors (Benor et al. 2020). Taken together, there is clear potential for tumors to contain mixtures of cells that express high and low (or no) levels of mutant p53 (Fig. 4).

While the impact of contacts between tumor cells that differ in p53 status has not been widely considered, emerging evidence suggests these interactions may have a role during oncogenesis. In mouse epithelium, cells expressing the p53-R273H mutant were eliminated from p53-null epithelia, as they were from p53 WT epithelia, clearly indicating that mutant p53 shows functions that are different from loss of p53 and, in this case, lead to the retention of the loser phenotype (Watanabe et al. 2018). An interesting form of cell competition results from entosis, a process by which winning cells can engulf surrounding loser cells (Overholtzer et al. 2007). In this case, cancer cells expressing mutant p53 were winners when competing with p53-null cells. Mixtures of isogenic p53-null and p53 R273H-expressing human carcinoma cells showed that p53-null cells were significantly more likely to be engulfed by mutant p53 cells. Further investigation revealed that mutant p53 cells were better at surviving the replication stress imposed by engulfing a cell, due to their ability to activate CHK1 and so persist through subsequent abnormal cell divisions such as tripolar mitosis. The resulting genomic instability in the daughters of engulfing cells led the authors to postulate that this process may act as a driver of intratumoral genetic heterogeneity. Interestingly, the presence of “cell-in-cell” structures was found to be highest in tumors with heterogeneous p53 expression and correlate with poor disease outcome and recurrence (Mackay et al. 2018). Parenthetically, engulfment of surrounding cells may also provide metabolic support for cancer cells under nutrient-limiting conditions (Fais and Overholtzer 2018).

## Conclusions

p53 is well established as a critical node in the cell's stress detection network, and it is therefore not surprising that p53 is implicated in determining cell fate in response to stresses imposed by other cells. The molecular mechanisms of mutant p53 action are still being debated, with the evidence for increased cancer-promoting capacity balanced by recent evidence that, in some tissues, mutant p53 may even function to suppress tumorigenesis (Kadosh et al. 2020). In addition, more attention is being given to how p53 mutations affect a cell's response to the presence of other cells within their surrounding environment at all stages of cancer development. The multitude of mutations detected in apparently normal human epithelia attests to the general robustness of tumor-suppressive processes, and there is a growing appreciation of the importance of interactions between cells in controlling or enhancing malignant progression. Various functions of WT and mutant p53 are clearly implicated in determining how cancer cells affect and respond to their environment, although much remains to be learned. Different activities of WT p53 and various cancer-associated p53 mutations, as well as heterogeneity in the levels of mutant p53 within tumors, provides added complexity. Despite these challenges, our appreciation of these functions of p53 can only serve to open up more avenues for treatment and improve our targeting of therapeutics.
